# Supplementary material for: Retinalysis-Vascx: An Explainable Software Toolbox for the Extraction of Retinal Vascular Biomarkers From Color Fundus Images
Source: Transl Vis Sci Technol. 2026 Sep 21;15(9):11. doi: 10.1167/tvst.15.9.11 (PMC13614130; doi:10.1167/tvst.15.9.11)
Supplement: Supplementary file 1 [file tvst-15-9-11_s001.pdf]

Appendix A. Optional biomarker arguments

Table 2. VascX biomarker optional arguments and description of their role in computation.

Biomarker Name	Optional Arguments
**BifurcationAngles**	- **delta** (20): Distance in pixels from the bifurcation where outgoing branch directions are sampled.<br>- **max_angle** (135): Maximum bifurcation angle, in degrees, kept before aggregation.<br>- **min_bifurcations** (3): Minimum number of valid bifurcations required to return a value.<br>- **spline_error_fraction** (0.05): Spline fitting tolerance used when estimating branch directions.<br>- **aggregator** (*mean*): Callable used to combine per-bifurcation angles.
**Caliber**	- **min_numpoints** (10): Minimum number of skeleton points a segment must have to be included.<br>- **aggregator** (*median*): Callable used to combine per-segment median diameters. Can also be *LengthWeightedAggregator*.<br>- **spline_error_fraction** (0.05): Spline fitting tolerance used for diameter estimation and length weighting.
**CRE**	- **CREMode** (*CREMode.Temporal*): Vessel-selection mode around the disc. Options are *Temporal*, *Nasal*, or *Full*.<br>- **max_vessels** (6): Maximum number of largest intersecting vessels kept at each circle.<br>- **hemifield** (*None*): Optional superior or inferior hemifield restriction.<br>- **min_circles** (6): Minimum number of valid circles required to return a value.<br>- **inner_circle** (1.0): Inner sampling radius in optic-disc-diameter multiples.<br>- **outer_circle** (1.5): Outer sampling radius in optic-disc-diameter multiples.<br>- **num_circles** (6): Number of concentric circles sampled between the inner and outer radii.<br>- **spline_error_fraction** (0.05): Spline fitting tolerance used for circle intersections and caliber estimation.
**Sparsity**	- **mode** (*SparsityMode.MEAN*): Sparsity summary mode. Options are *MEAN* and *MAX*.<br>- **normalize** (*True*): Whether to divide distances by the disc-fovea distance.

**TemporalAngle**	- **inner_circle** (*2 / 3*): Inner sampling radius as a fraction of the disc-fovea distance.<br>- **outer_circle** (*2 / 3 + 4 * 0.03*): Outer sampling radius as a fraction of the disc-fovea distance.<br>- **num_circles** (5): Number of concentric circles sampled between the inner and outer radii.<br>- **spline_error_fraction** (0.05): Spline fitting tolerance used for circle intersections and pair geometry.
**Tortuosity**	- **mode** (*TortuosityMode.Segments*): Whether tortuosity is computed on segments or resolved vessels.<br>- **measure** (*TortuosityMeasure.Distance*): Tortuosity definition. Options are *Distance*, *Curvature*, and *Inflections*.<br>- **length_measure** (*LengthMeasure.Splines*): Length source used for distance tortuosity.<br>- **min_numpoints** (25): Minimum number of skeleton points required for inclusion.<br>- **max_segment_len** (*None*): Optional maximum split length, as a fraction of disc-fovea distance, used only for segment-mode distance tortuosity.<br>- **max_tortuosity** (*None*): Optional upper cutoff for distance tortuosity. If left as *None*, the class uses *1.5* for distance tortuosity and no cutoff otherwise.<br>- **aggregator** (*median*): Callable used to combine per-segment or per-vessel tortuosity values. Can also be *LengthWeightedAggregator*.<br>- **spline_error_fraction** (*None*): Spline fitting tolerance used before tortuosity computation. If left as *None*, the class uses *0.05* for distance tortuosity and *0.25* for curvature or inflection tortuosity.
**VascularDensity**	- No constructor arguments besides *grid_field*.

Appendix B. Test-retest reproducibility analysis

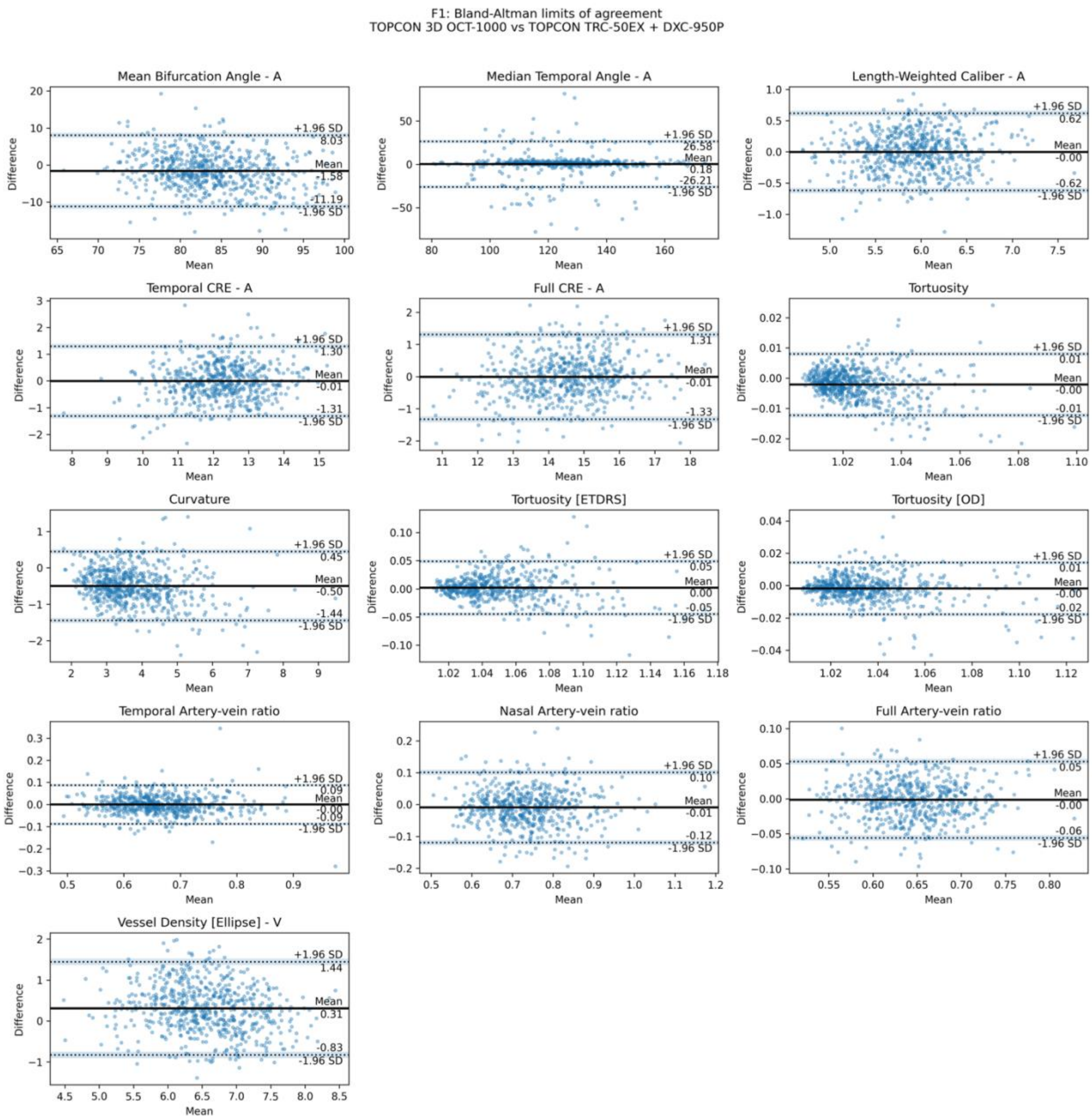

Figure 11. Bland-Altman plots on pairs of images of the same eye by different CFI devices, for optic-disc-centered (F1) images by the Topcon 3D OCT-1000 and Topcon TRC-50EX imaging devices.

Appendix C. Biomarker sensitivity analysis

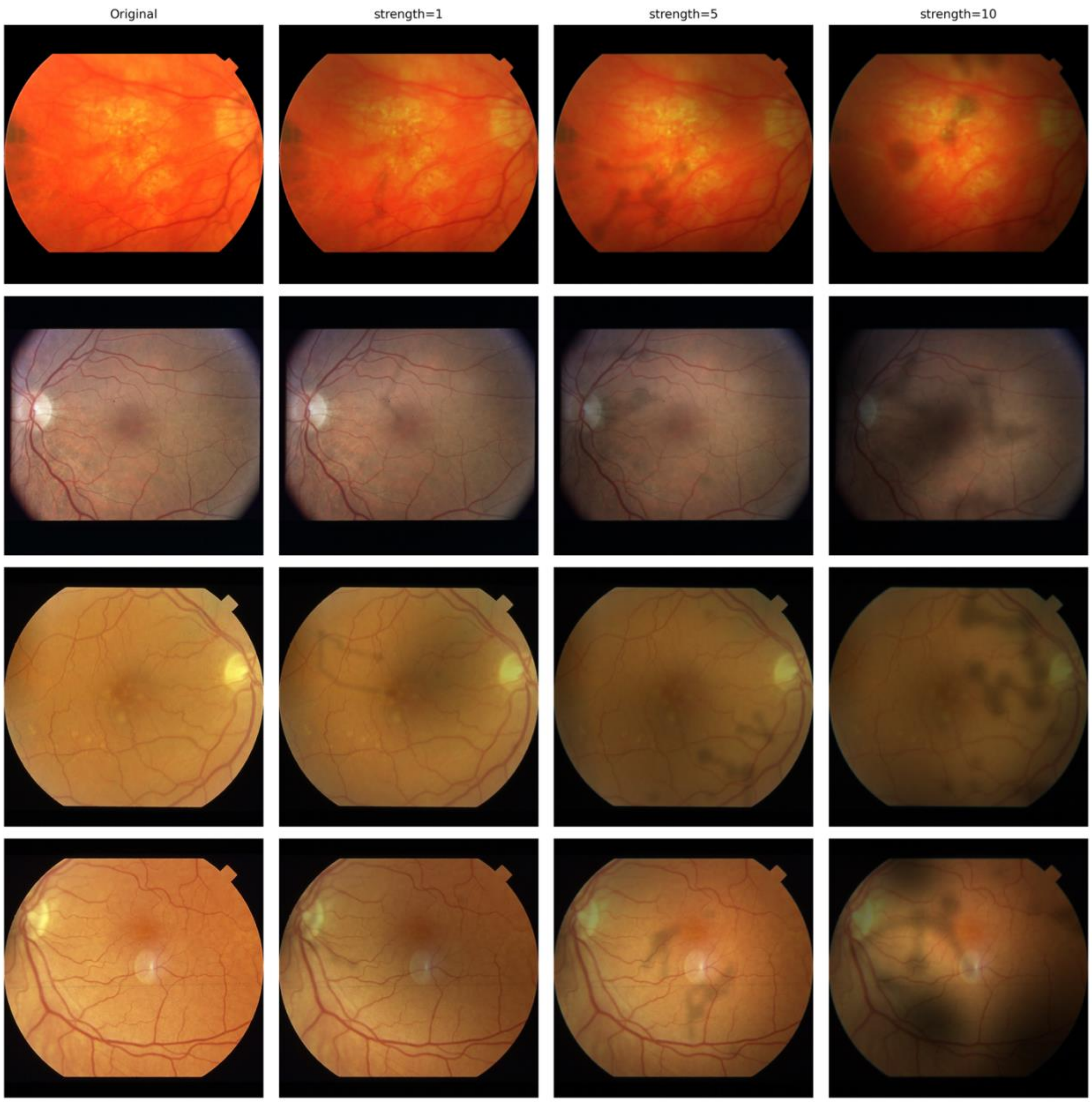

Figure 12. Sample images from our CFI augmentation pipeline used in the biomarker sensitivity analysis. The left-most column shows the four original samples. Each row displays an image with perturbations of different strengths applied to it.

Appendix D

The VascX Research Consortium (in alphabetical order):

Ciara Bergin 3; Sven Bergmann 1,2,4; Michael Beyeler 1,2,5; Dennis Bontempi 1,2; Sacha Bors 1,2; Leah Böttger 1,2; Bogdan Draganski 5,6,7; Adham Elwakil 3,8; Györgyi V. Hamvas 9,10; Janna Hastings 11,12; Ilaria Iuliani 1,2; Caroline C.W. Klaver 13,14,15,16; Ihor Kuras 6,7; Bart Liefers 13,14; Ilenia Meloni 3,8; Sofia Ortin Vela 1,2; David Presby 1,2; Ian Quintas 1,2; José Vargas Quiros 13,14,; Marc Schindewolf 9,10; Reinier O. Schlingemann 3,17; Mattia Tomasoni 3,8; Olga Trofimova 1,2.

1 Department of Computational Biology, University of Lausanne, Lausanne, Switzerland

2 Swiss Institute of Bioinformatics, Lausanne, Switzerland

3 Department of Ophthalmology, University of Lausanne, Fondation Asile des Aveugles, Jules Gonin Eye Hospital, Lausanne, Switzerland.

4 Department of Integrative Biomedical Sciences, University of Cape Town, Cape Town, South Africa

5 Insel University Hospital Bern, Switzerland

6 Department of Neurology, Max Planck Institute for Human Cognitive and Brain Sciences, Germany

7 Department of Clinical Neuroscience, Lausanne University Hospital and University of Lausanne, Switzerland

8 Platform for Research in Ocular Imaging, Fondation Asile des Aveugles, Jules Gonin Eye Hospital, Lausanne, Switzerland.

9 Inselspital, Bern University Hospital, University of Bern, Switzerland.

10 Department for BioMedical Research, Bern University Hospital, University of Bern, Switzerland.

11 Institute for Implementation Science in Health Care, Faculty of Medicine, University of Zurich, Zürich, Switzerland

12 School of Medicine, University of St Gallen, St. Gallen, Switzerland

13 Department of Ophthalmology, Erasmus University Medical Center, Rotterdam, The Netherlands.

14 Department of Epidemiology, Erasmus University Medical Center, Rotterdam, The Netherlands.

15 Department of Ophthalmology, Radboud University Medical Center, Nijmegen, the Netherlands.

16 Institute of Molecular and Clinical Ophthalmology, University of Basel, Switzerland.

17 University Medical Centres, Amsterdam, The Netherlands.
